# Investigating barriers to the protective efficacy provided by rotavirus vaccines in African infants

**DOI:** 10.1371/journal.pmed.1003721

**Published:** 2021-08-10

**Authors:** Julie E. Bines

**Affiliations:** 1 Department of Paediatrics, University of Melbourne, Parkville, Victoria, Australia; 2 Department of Gastroenterology and Clinical Nutrition, Royal Children’s Hospital, Parkville, Victoria, Australia; 3 Enteric Diseases Group, Murdoch Children’s Research Institute, Parkville, Victoria, Australia

## Abstract

Julie Bines discusses an accompanying study by Sheila Isanaka and colleagues on nutrient supplementation and immune responses to rotavirus vaccination.

The introduction of rotavirus vaccines into the national immunization programs globally has made a major impact on diarrhea-related hospitalizations and deaths. By 2020, 107 countries had introduced rotavirus vaccines, either nationally or regionally, including 52 countries in Africa and Asia eligible for funding through the Global Alliance for Vaccines and Immunization (Gavi) [1]. This represents a major step toward reducing under 5-year child mortality, the impact of rotavirus disease on child health, and the economic burden on families and the healthcare system. A remaining challenge is the lower vaccine protective efficacy observed in children in low- and middle-income countries (LMICs) where the mortality and hospitalizations due to severe rotavirus disease still occur [1]. The role of nutrition in influencing the immune response to a rotavirus vaccine is the focus of the accompanying paper by Isanaka and colleagues published in this issue of *PLOS Medicine* [2].

Understanding why over 87% of children vaccinated with a rotavirus vaccine in low child mortality countries are protected against severe rotavirus disease compared to approximately 44% (27% to 59%) of children in high child mortality countries is not well understood [3]. As an orally administered vaccine, initial focus has been on factors that could neutralize the live vaccine virus within the gut lumen. Most rotavirus vaccines are administered in a buffered formulation to reduce the risk of neutralization of the vaccine virus by gastric acid [4]. In early clinical trials, fasting prior to vaccination was applied in an effort to reduce the potential impact of breast milk antibodies. This is now not considered necessary [5]. A difference in the gut microbiome in infants from high-income and LMICs has been observed, although the administration of a probiotic did not result in improved rotavirus vaccine immunogenicity [6,7]. Rotaviruses use histo-blood group antigens present on the gut epithelial surface in the initial phase of virus attachment and cellular entry [8]. It has been proposed that population variability in histo-blood group antigen phenotype, specifically Lewis and secretor status, may explain the genotype diversity of rotavirus between regions and the responses observed to live oral rotavirus vaccines that may be VP4 [P] genotype dependent [8].

Childhood malnutrition is associated with reduced immune responses to a range of infections, and in the immune response to vaccines, including rotavirus vaccines [9]. Macro and/or micronutrient deficiencies have been linked to a range of abnormalities in T and B cell function, mucosal immunity, cytokine production, and responses [9]. However, there are limited data on the impact of maternal nutritional supplements on the immune responses following vaccination of their infants. Isanaka and colleagues’ cluster-randomized study was nested within a double-blind, placebo control vaccine efficacy trial. It evaluated the effect of 3 different maternal nutritional supplements on serum antirotavirus immunoglobulin A (IgA) seroconversion following administration of 3 doses of the oral rotavirus vaccine Rotasiil (G1, G2, G3, G4, G9) in infants in Niger [2]. The daily supplements were commenced prior to 30 week’s gestation in a population of women at risk of macro- and micro-nutrient malnutrition, although maternal anthropometry and micronutrient status before and after supplementation is not reported. The supplement options included the “standard of care” iron−folic acid (IFA) supplement, a multi-micronutrient (MMN) supplement at levels at or double the US recommended dietary allowance for pregnant women, or the same MMN supplement with an additional energy and protein component. As all groups received a supplement, this study was designed to provide a comparison between supplement groups rather than a comparison with no supplement. Across all supplement groups, the serum antirotavirus IgA seroconversion following administration of 3 doses of Rotasiil was modest at 39.6% and only 10% greater than that observed in the placebo group (29.0%). The rate of seroconversion did not differ between supplement groups, although serum IgA geometric mean titres were not reported. In similar study in The Gambia, an enhanced antibody response to the diphtheria-tetanus-pertussis (DPT) vaccine was observed in the infants of mothers who had received a prenatal MMN and protein−energy supplement, when compared to those who received the “standard of care” iron−folate supplement [10]. Of note, the supplement used in The Gambia study contained more energy and protein when compared to the MMN plus energy and protein used in this study in Niger (The Gambia study versus Niger study; energy [kcal]: 746 versus 237; protein [grams]: 20.8 versus 5.2; lipids [grams]: 52.6 versus 20). Whether the differences reported in vaccine immune responses between these 2 studies reflect these differences in the composition of the supplement, differences specific to the vaccine (DPT versus rotavirus vaccine), study sample size or characteristics of the study population requires further study.

Rotavirus vaccines save lives and prevent hospitalizations due to rotavirus disease in children. Efforts to improve the level of protection provided by rotavirus vaccines, particularly in LMICs, have the potential to maximize the impact on these vaccines on global child health. Improving the nutritional status of infants through the provision of macro- and micro-nutrient supplements to pregnant mothers in high-risk populations may optimize immune responses to rotavirus vaccines; however, the specific composition of the prenatal supplement requires further investigation.

## Abbreviations

DPT: diphtheria-tetanus-pertussis
Gavi: Global Alliance for Vaccines and Immunization
IFA: iron−folic acid
IgA: immunoglobulin A
LMICs: low- and middle-income countries
MMN: multi-micronutrient

## References

[1] ViewHub [Internet]. [cited 2020 Dec 29]. Available from: https://view-hub.org/map/?set=current-vaccine-intro-status&group=vaccine-introduction&category=rv

[2] Isanaka S, Garba S, Plikarytis B, McNeal MM, Guindo O, Langendorf C, et al. Immunogenicity of an oral rotavirus vaccine administered with prenatal nutritional support in Niger: a cluster randomized clinical trial. PLoS Med. Forthcoming [2021]. 10.1371/journal.pmed.1003720 PMC8354620 34375336

[3] Clark A, van Zandvoort K, Flasche S, Sanderson C, Bines J, Tate J, et al. Efficacy of live oral rotavirus vaccines by duration of follow-up: a meta-regression of randomised controlled trials. J Infect Dis. 2019;19:717–27. doi: 10.1016/S1473-3099(19)30126-4 31178289 PMC6595176

[4] Kumar P, Pullagurla SR, Patel A, Shukla RS, Bird C, Kumru OS, Hamidi A, et al. Effect of formulation variables on the stability of a live, rotavirus (RV3-BB) vaccine candidate using in vitro Gastric Digestion models to mimic oral delivery. J Pharm Sci. 2020. doi: 10.1016/j.xphs.2020.09.047 33035539 PMC7815322

[5] Ali A, Kazi AM, Cortese MM, Fleming JA, Moon SS, Parashar U, et al. Impact of Withholding Breastfeeding at the Time of Vaccination on the Immunogenicity of Oral Rotavirus Vaccine—A Randomized Trial. PLoS ONE. 2015;10(6):e0127622. doi: 10.1371/journal.pone.0127622 26035743 PMC4452702

[6] Harris VC, Armah G, Fuentes S, Korpela KE, Parashar U, Victor JC, et al. Significant Correlation Between the Infant Gut Microbiome and Rotavirus Vaccine Response in Rural Ghana. J Infect Dis. 2017;215:34–41. doi: 10.1093/infdis/jiw518 27803175 PMC5225256

[7] Lazarus RP, John J, Shanmugasundaram E, Rajan AK, Thiagarajan S, Giri S, et al. The effect of probiotics and zinc supplementation on the immune response to oral rotavirus vaccine: A randomized, factorial design, placebo-controlled study among Indian infants. Vaccine. 2018;36(2):273–9. doi: 10.1016/j.vaccine.2017.07.116 28874323 PMC12001858

[8] Boniface K, Byars S, Cowley D, Kirkwood CD, Bines JE. Human Neonatal Rotavirus Vaccine (RV3-BB) produces vaccine take irrespective histo-blood antigen status. J Infect Dis. 2020;2221(7):1070–81. doi: 10.1093/infdis/jiz333 31763671 PMC7075413

[9] Rytter MJH, Kolte L, Briend A, Friis H, Christensen VB. The Immune System in Children with Malnutrition—A Systematic Review. PLoS ONE. 2014;9(8):e105017. doi: 10.1371/journal.pone.0105017 25153531 PMC4143239

[10] Okala SG, Darboe MK, Sosseh F, Sonko B, Faye-Joof T, Prentice AM, Moore SE. Impact of nutritional supplementation during pregnancy on antibody responses to diphtheria-tetanus-pertussis vaccination in infants: A randomised trial in The Gambia. PLoS Med. 2019;16(8):e1002854. doi: 10.1371/journal.pmed.1002854 31386660 PMC6684039

